# *Dendrocerus mexicali* (Hymenoptera, Ceraphronoidea, Megaspilidae): Novel antennal morphology, first description of female, and expansion of known range into the U.S.

**DOI:** 10.3897/zookeys.569.6629

**Published:** 2016-02-24

**Authors:** Kyle N. Burks, István Mikó, Andrew R. Deans

**Affiliations:** 1 Pennsylvania State University, Department of Entomology, University Park, 16802, United States of America

**Keywords:** *Dendrocerus*, morphology, taxonomy, flabellate, ramose, antennae

## Abstract

*Dendrocerus mexicali* has been described by Paul Dessart from a single male specimen collected in Mexico. Using 87 newly identified specimens we expand the known range to include the Southwestern United States and Florida, provide an expanded description of the species, and provide the first record of the female. We also use confocal laser scanning microscopy and *in vitro* hydrostatic pressure changes to investigate the functional morphology of apparently unique basally flexible antennal branches.

## Introduction

Ceraphronoidea (Hymenoptera) is a widespread superfamily of parasitoid wasps comprised of two extant families: Ceraphronidae and Megaspilidae. Little is known about the biology of Ceraphronidae, but there are quite a few host records for Megaspilidae, especially for the genus *Dendrocerus* Ratzeburg, 1852 (Fergusson 1980; Dessart 1999).

Host records suggest that *Dendrocerus* parasitizes a broad range of orders, including Hemiptera, Neuroptera, Coleoptera, Diptera, Hymenoptera, (Fergusson 1980, Dessart 1995). Many of its hosts are predators or parasitoids of non-heteropteran Hemiptera, especially of aphids (Aphididae) (Fergusson 1980; Dessart 1995). Based on host records, some species are specialists, while others are generalists, and while a few may be primary parasitoids, many *Dendrocerus* are hyperparasitoids (Fergusson 1980). *Dendrocerus carpenteri*, which has a very broad host range, has been recorded as being a secondary, tertiary, and even quaternary parasitoid (Fergusson 1980; Haviland 1920).

*Dendrocerus mexicali* was first described from a single male specimen collected on wild mustard in Mexicali, Mexico (Dessart 1999). Little is known about its natural history. The female has never been described and the host relationships of *Dendrocerus mexicali* remain unknown.

**Figure 1.**
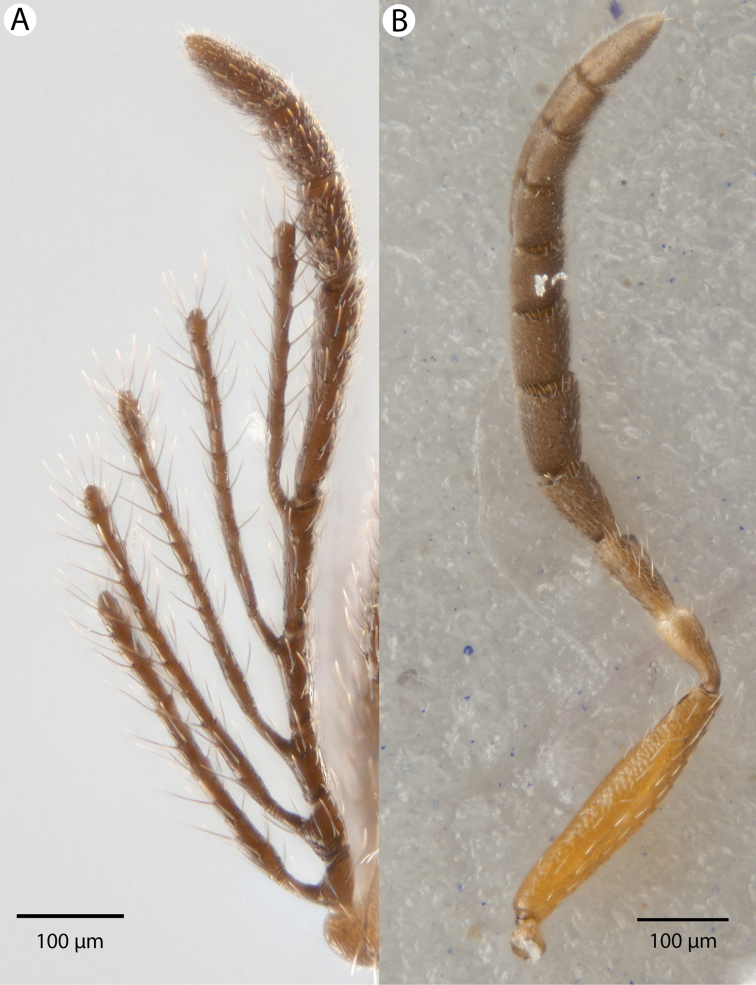
Bright field images of *Dendrocerus mexicali* antenna. **A** male, pedicel and ramose flagellomeres **B** female, scape, pedicel, and clavate flagellomeres.

*Dendrocerus mexicali*, like other *Dendrocerus* species of the *halidayi* species group, have antennae with long flagellar projections (flagellomeres are “branched” or “ramose”). The antennae of the male *Dendrocerus mexicali* is perhaps its most distinguishing feature (Dessart 1999). While branched antennae are not uncommon across Hymenoptera, including *Dendrocerus*, the base of each flagellar process is wrinkled and is lighter than the flagellomere or the process (Figure 1A). The function of this region is unknown, and even Dessart was not sure if it was an artifact of preservation (Dessart 1999). One of the aims of this study is to investigate the function of this region.

## Methods

All specimens are point-mounted and air-dried. Specimens are deposited in the University of Central Florida Arthropod Collection (UCFC) (18 males and 5 females), the Canadian National Collection of Insects, Arachnids, and Nematodes (CNC) (9 males and 55 females) and Pennsylvania State University Collection Frost Entomological Museum (PSUC_FEM) identifier. All figures, OWL files, and supplementary files are available on Figshare (https://dx.doi.org/10.6084/m9.figshare.2063586.v1).

### Dissections

Dissections were performed in glycerol or on Blue-Tack (Bostik, Inc., Wauwatosa, WI, USA) using number 2 insect pins and an Olympus SZX16 stereomicroscope, with an Olympus SDF PLAPO 1XF objective (115×) and an Olympus SDF PLAPO 2XPFC objective (230× magnification).

### Confocal laser scanning microscopy (CLSM)

CLSM was used to image the male antenna and genitalia. Dissected male *Dendrocerus mexicali* antennae and genitalia were placed in a droplet of glycerol between two no. 1.5 coverslips with a small amount of Blue-Tack as a spacer (Mikó and Deans 2013). Specimens were examined with an Olympus FV10i Confocal Laser Scanning Microscope. The antenna was imaged using three excitation wavelengths: 405 nm, 473 nm, and 559 nm. Autofluorescence was detected and assigned a pseudocolor using three channels with emission ranges of 420–520 nm (blue), 490–590 nm (green), and 570–670 nm (red), respectively. Volume rendered micrographs and media files were created in ImageJ (Schneider et al. 2012) using maximum intensity. The genitalia was imaged using two excitation lasers of 631 nm and 499 nm. Two channels were used to detect emissions of 647 nm (green) and 520 nm (red), respectively.

### Bright field images

Bright field images were taken using an Olympus ZX41 compound microscope with an attached Olympus DP71 digital camera. Images were stacked and aligned using Zerene Stacker Version 1.04 Build T201404082055.

### Antenna coiling experiment

Following the methods described by Steiner et al. (2010), we removed the antenna from one specimen stored in glycerol and one dried and pinned specimen. Both were macerated in 10% KOH for 10 minutes, and then stored in 80% ethanol overnight. We then placed the antenna in 100% ethanol for 10 minutes before transferring to distilled water.

### mx autogenerated description

Specimen data, specimen images, OTU concepts and phenotypes expressed in natural language were compiled in mx (http://purl.org/NET/mx-database) and the description and material examined sections of this article were automatically generated from this software. Morphological terminology in the description and diagnosis are linked to classes in phenotype-relevant ontologies (Hymenoptera Anatomy Ontology (HAO), Phenotypic Quality Ontology (PATO), Biospatial Ontology (BSPO), OBO Relation Ontology (RO), Ontology for Biomedical Investigations (OBI), and Information Artifact Ontology (IAO); all of which are available at http://www.ontobee.org/).

### Semantic statement generation

Phenotype descriptions expressed as semantic statements (Suppl. material 1, 2) were generated using Protégé Version 5.0.0 (Build beta 17) following Balhoff et al. (2013) and Mikó et al. (2014). Semantic statements for the taxonomic treatment of *Dendrocerus mexicali* are available as supplementary OWL files (Suppl. material 3) and were deposited on Figshare (https://dx.doi.org/10.6084/m9.figshare.2063586.v1).

### Abbreviations used

CSB: cephalic size, HH: head height, EH: eye height, HL: head length, HW: head width, IOS: interorbital space, OOL: ocular ocellar length, LOL: lateral ocellar length, POL: posterior ocellar length, MscL: median mesoscutal line, AscW: anterior mesoscutal width, PscW: posterior mesoscutal width.

## Results

### Dendrocerus mexicali

Taxon classificationAnimaliaHymenopteraMegaspilidae

Dessart, 1999

Figures 1 , 2 , 3 , 4 , 5 , 6 , 7 , 8 , 9

#### Diagnosis.

**Figure 2.**
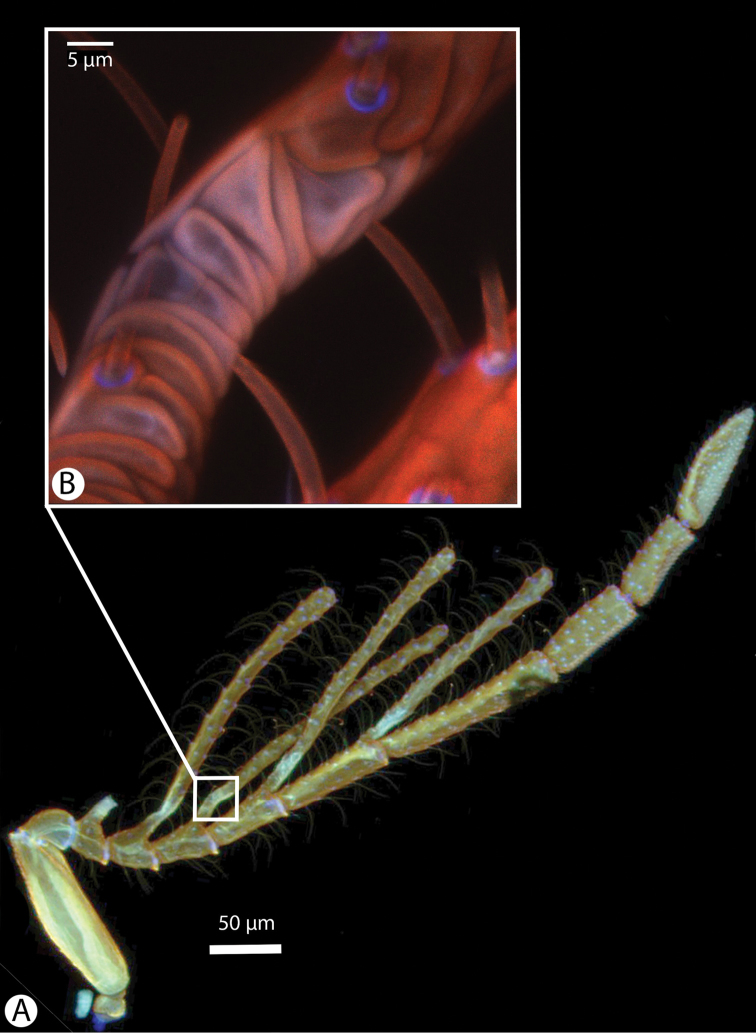
Confocal laser scanning microscopic images of male *Dendrocerus mexicali* antenna. **A** Antenna with the most basal branch (branch of 1st flagellomere) missing. Bluish area at base of branches indicates a high concentration of resilin; orange and red indicate sclerotized regions; green indicates softer, non-sclerotized regions **B** Magnified view of branch articulation. Purple and pink areas indicate high concentrations of resilin in the cuticle; blue indicates areas of extremely high resilin concentration; red indicates strongly sclerotized regions.

**Figure 3.**
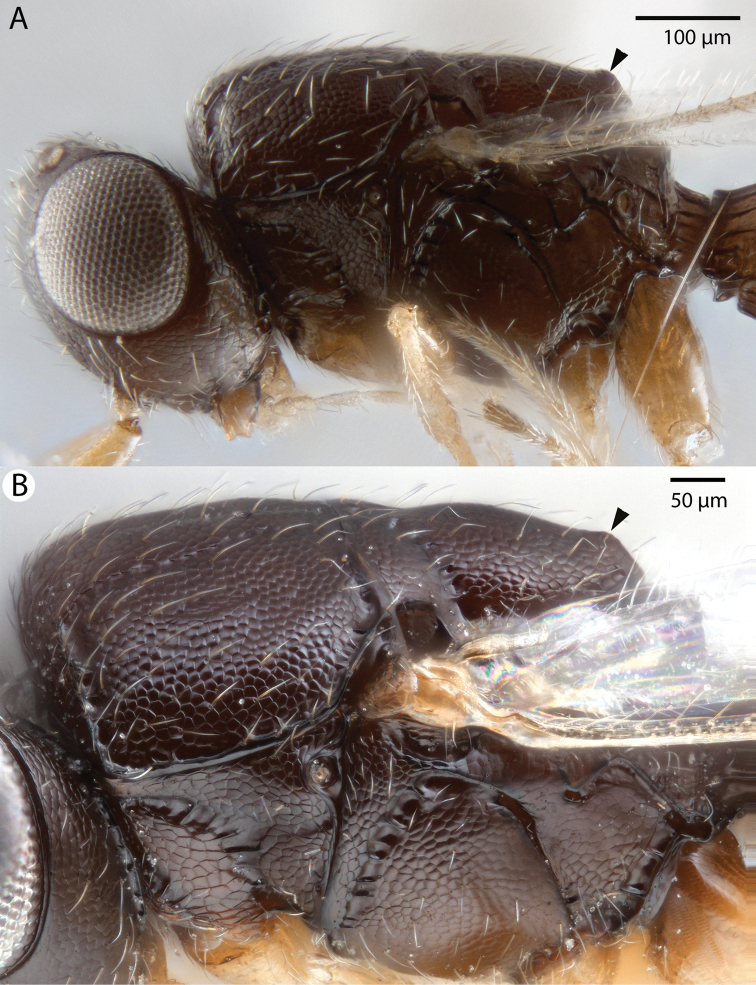
Bright field images of *Dendrocerus mexicali* mesosoma, lateral view. Arrows indicate the location of a mucro. **A** Male **B** Female.

**Figure 4.**
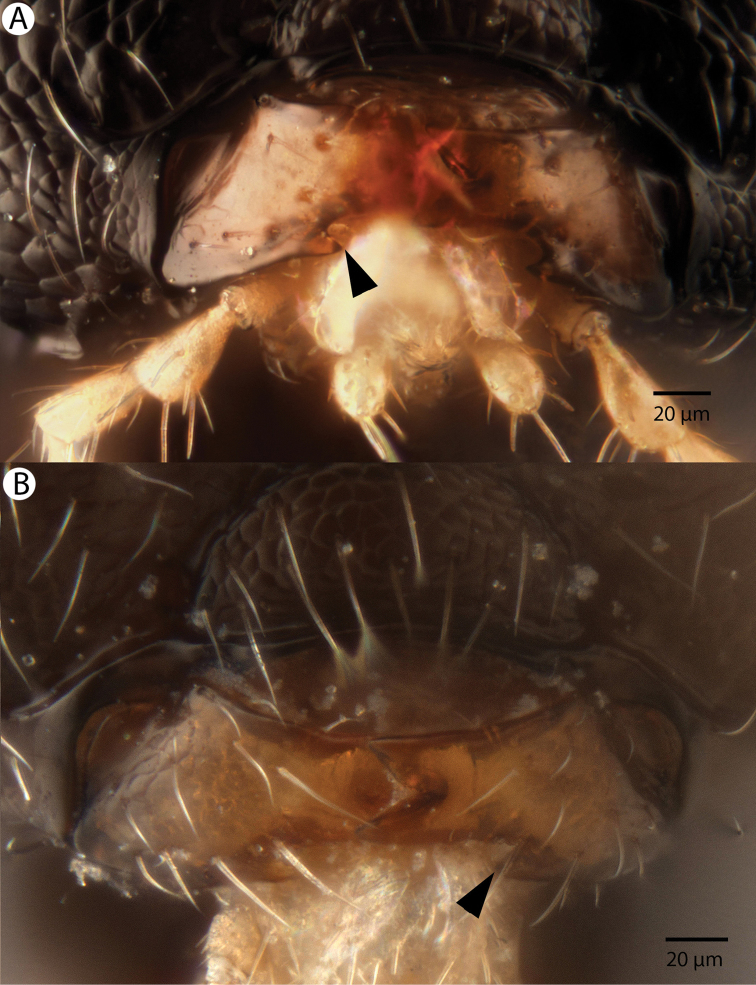
Bright field images of *Dendrocerus mexicali* mouthparts, anterior view. Arrows indicate the location of mandibular lancea. **A** Male **B** Female.

**Figure 5.**
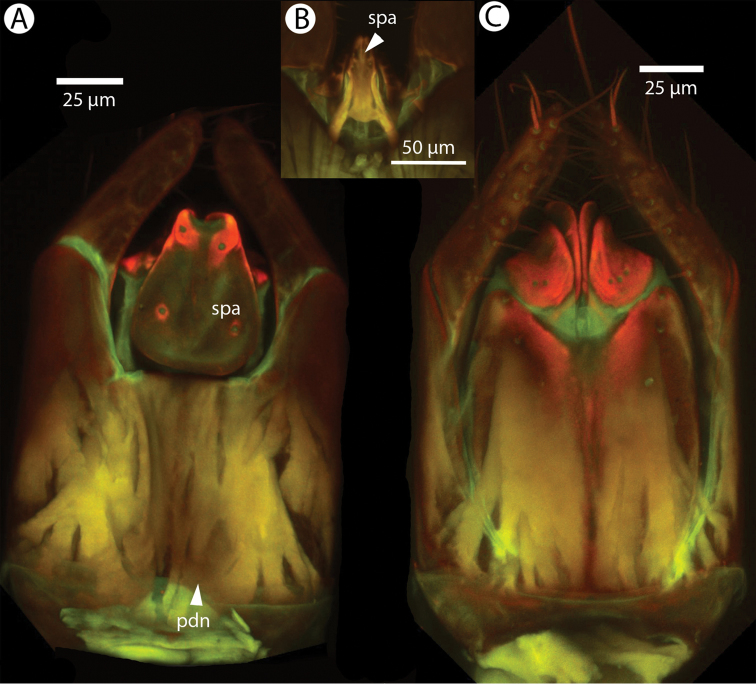
Confocal laser scanning microscopic images of male genitalia; spa=Sensillar plate of the aedeagus. **A** *Dendrocerus mexicali* dorsal view **B** *Dendrocerus ramicornis* dorsal view for spa size comparison **C** *Dendrocerus mexicali* ventral view.

Male flagellomeres have projections with flexible, wrinkled regions at base (Figures 1A and 2). Both males and females have a blunt posteromedian process of the mesoscutellum, called a mucro, that is less sharp than that of *Dendrocerus koyamae* (Figure 3). Both males and females have mandibular lancea (Figure 4). The sensillar plate of the male aedeagus is strongly sclerotized and greatly enlarged compared to all other described Megaspilidae (Figure 5).

#### Description.

Body length universal: 1.4–1.7 mm (n=10). Color hue pattern: antenna, legs, mouthparts ochre; rest of body dark brown. Color intensity pattern: flagellomeres and their branches darker than scape and pedicel. scape and pedicel same as legs. Cephalic size (csb): Mean: 400–500μm. head height (lateral view) vs eye height (anterior view): HH:EHf=1.4–1.8 (n=5). head height vs. head length: HH:HL=1.4–1.8 (n=5). head width vs. interorbital space: HW/IOS=1.8–2.0 (n=5). head width vs. head height: HW/HH=1.2–1.4 (n=5). Male OOL:LOL: OOL/LOL=0.75–1.0 (n=2). Male OOL:POL: OOL/POL=0.24–0.43 (n=2). Female OOL:LOL: OOL 0.625–0.75× as long as LOL (n=3). Anterior ocellar fovea shape: fovea not extended ventrally to the dorsal margin of antennal scrobe. occipital carina sculpture: smooth. submedial flange of occipital carina count: absent. median flange of occipital carina count: absent. preoccipital carina and occipital carina structure: the occipital carina extends ventrally to the oral foramen with the preoccipital carina present on the vertex, but not extendinig ventrally along the gena. preoccipital carina count: present . preoccipital carina shape: present medially, absent laterally to lateral ocelli. preoccipital lunula count: present. preoccipital furrow count: present. preoccipital furrow anterior end: preoccipital furrow ends inside ocellar triangle. dorsal margin of occipital carina vs dorsal margin of lateral ocellus in lateral view: occipital carina is ventral to lateral ocellus in lateral view. Transversely reticulate region on frons count: absent. Rugose region on frons count: absent. facial pit count: facial pit present. intertorular carina count: present. Ventral margin of antennal rim vs dorsal margin of clypeus: not adjacent. Median region of intertorular area shape: flat. subtorular carina count: absent. torulo-clypeal carina count: present. supraclypeal depression count: present. supraclypeal depression structure: present medially, inverted U-shaped. antennal scrobe count: absent. flagellomere shape (male): branched. scape length relative to length of F1+F2 (male): longer or equal. 6th male flagellomere length vs. width, “sensillar” view : elongate, more than 2× as long as wide. flagellomere branch count: 5 branches. Branch of male flagellomere 5 length compared to flagellomere 6: Longer than length of flagellomere 6. Branch of male flagellomere 5 length compared to flagellomere 5: Longer than length of flagellomere 5. flagellomere 6 length compared to flagellomeres 7+8: Equal to the length of flagellomere 7+8. sensillar patch of the male flagellomere pattern: F6—F9. Basal resilin-rich area of male antennal branches count: present. Female first flagellomere length vs pedicel : F1 as long as pedicel (1.0–1.1) (n=3). Female ninth flagellomere length: F9 less than F7+F8. Mandibular tooth count: 2. mandibular lancea count: present. ventrolateral invagination of the pronotum count: present. atrium of the anterior thoracic spiracle size: as wide as distal trachea. notaulus posterior end location: adjacent to transscutal articulation. epicnemial carina count: complete. epicnemium posterior margin shape: anterior discrimenal pit absent; epicnemial carina straight. speculum ventral limit: extending ventrally of pleural pit line. sternaulus count: absent. Median mesoscutal line length vs anterior mesoscutal width: MscL/AscW=0.6–0.9 (n=5). anterior mesoscutal width vs. posterior mesoscutal width: AscW/PscW=0.9 (n=5). median mesoscutal sulcus posterior end: adjacent to transscutal articulation. axillular carina count: absent. posteromedian process of the mesoscutellum count: present. posteromedian process of the mesoscutellum shape: blunt. scutoscutellar sulcus vs transscutal articulation: adjacent. mesometapleural sulcus count: present. posterodorsal metapleural area shape: trapezoid. metapleural carina count: present. anteromedian projection of the metanoto-propodeo-metapecto-mesopectal complex count: absent. lateral propodeal carinae shape: inverted “V” (left and right lateral propodeal carinae are adjacent medially at their intersection with antecostal sulcus of the first abdominal tergum). lateral propodeal carina count: present. transverse line of the metanotum-propodeum vs. antecostal sulcus of the first abdominal tergum: adjacent sublaterally. Distal margin of male abdominal sternum 9 shape: convex. median conjunctiva of abdominal tergum 9 count: absent. Proximolateral corner of abdominal sternum 9 shape: blunt. proximodorsal notch of cupula count: absent. Gonostyle/volsella complex proximodorsal margin shape: with deep concavity medially. Submedian conjunctiva on distoventral margin of gonostyle/volsella complex: length (range of fusion of parossiculus/parossiculus complex from gonostipes): more than 4/5. apical parossiculal seta number: two. dorsal apodeme of penisvalva count: absent. distal projection of the penisvalva count: absent. sensillar plate of the aedeagus shape: Enlarged, about half as wide as the genitalia, and strongly sclerotized. carina limiting posteriorly antecosta count: present. distal projection of the parossiculus count: absent. dorsomedian conjunctiva of the gonostyle-volsella complex count: absent. cupula length vs. gonostyle-volsella complex length: cupula less than 1/2 the length of gonostyle-volsella complex in lateral view. parossiculus count (parossiculus and gonostipes fusion): absent (fused with the gonostipes). distoventral submedian corner of the cupula count: absent. harpe length: harpe shorter than gonostipes in lateral view.

**Figure 6.**
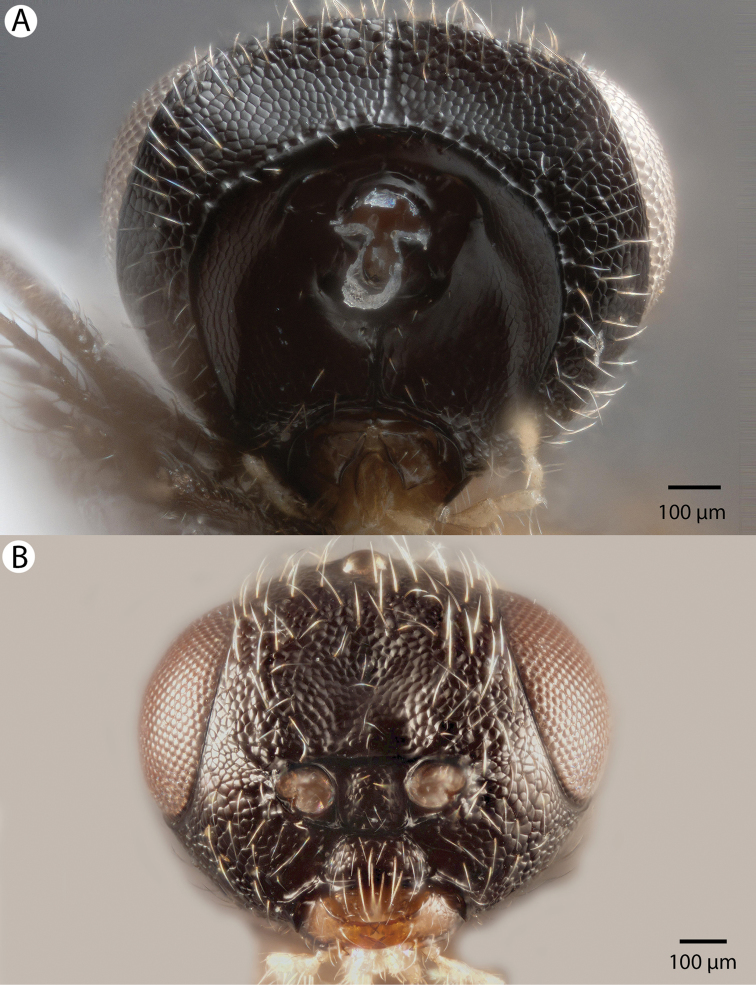
Bright field images of male Dendrocerus mexicali head. **A** Posterior view **B** Anterior view.

**Figure 7.**
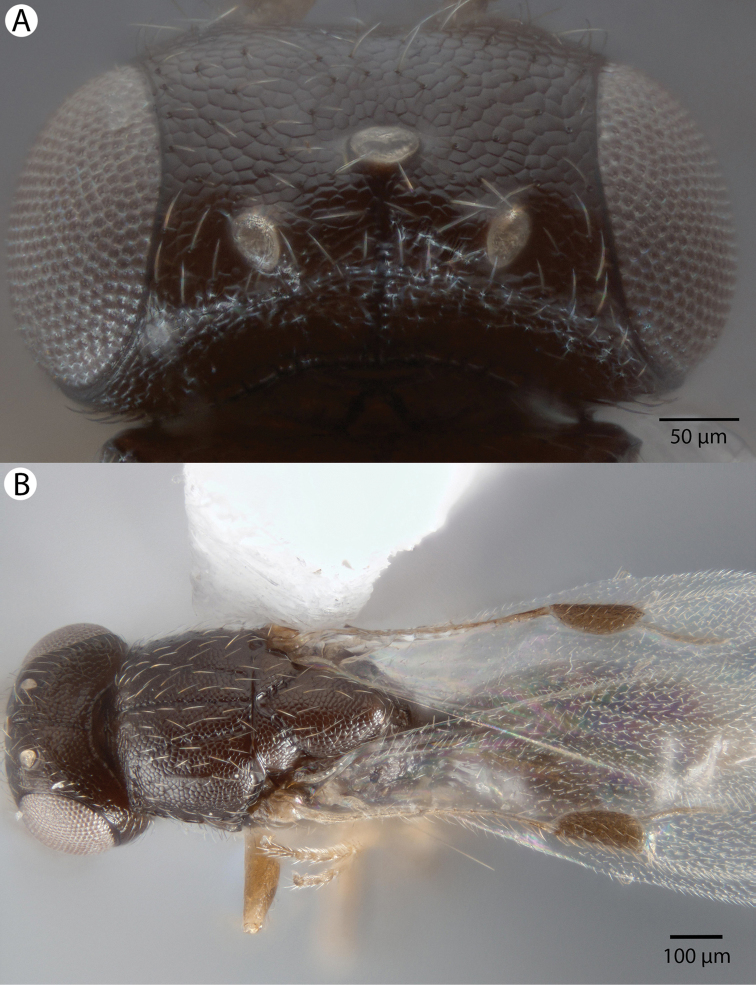
Bright field images of male *Dendrocerus mexicali*. **A** Dorsal view of head **B** Habitus; dorsal view.

**Figure 8.**
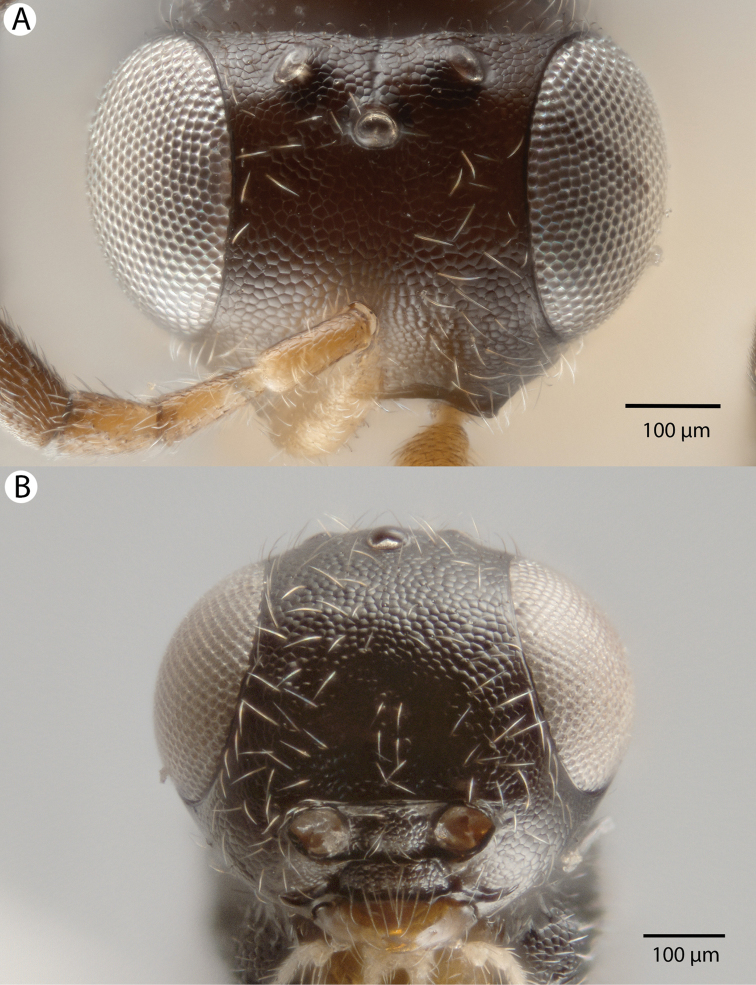
Bright field images of female *Dendrocerus mexicali* head. **A** Dorsal view **B** Anterior view.

**Figure 9.**
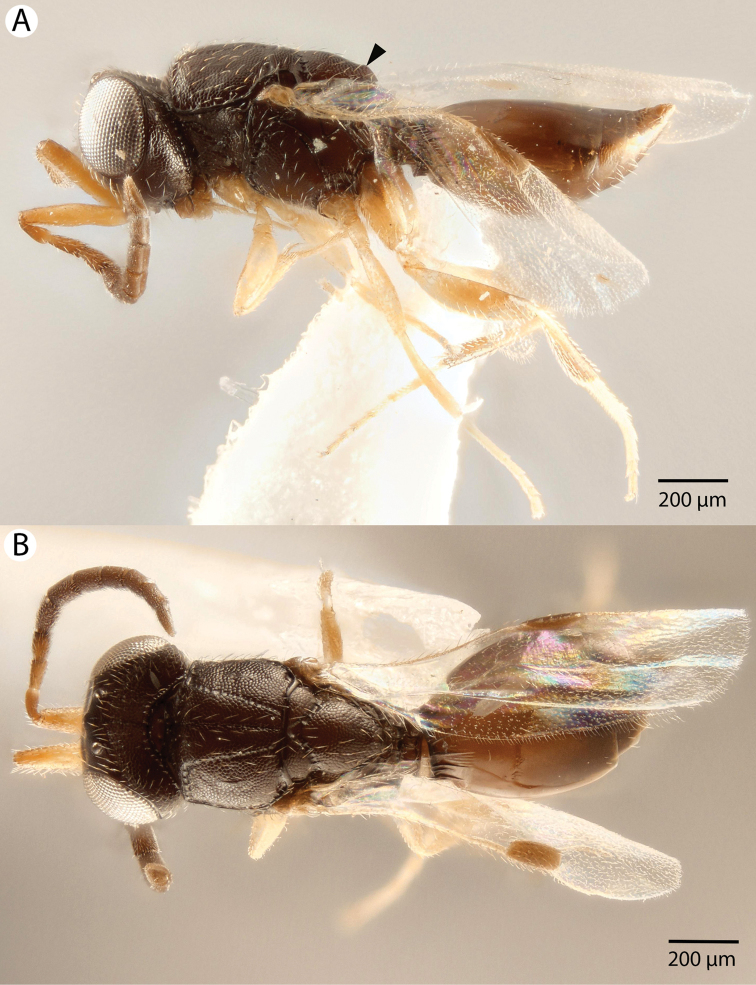
Bright field images of female *Dendrocerus mexicali* habitus. **A** Lateral view **B** Dorsal view.

#### Range.

Mexico (Mexicali), California, Arizona, Texas, and Florida.

#### Material examined.

Other material (60 females, 27 males): USA:Arizona:Santa Cruz Co.: 1 male. PSUC_FEM 86285 (PSUC). USA:California:Stanislaus Co.: 1 male. IM 5156 (UCFC). USA:Florida: 8 females, 13 males. PSUC_FEM 98899, 98907 (PSUC); IM 5106, 5165, 5214; PSUC_FEM 86151, 86166, 86366, 86370, 86384, 86443 (UCFC); PSUC_FEM 56350–56352, 56397–56403 (CNC). USA:Florida:Brevard Co.: 2 males. IM 5212; PSUC_FEM 86296 (UCFC). USA:Florida:Highlands Co.: 47 females, 6 males. PSUC_FEM 56353–56359, 56361-56396, 56404–56413 (CNC). USA:Florida:Orange Co.: 3 males. IM 5210–5211; PSUC_FEM 86137 (UCFC). USA:Florida:Polk Co.: 4 females, 1 male. IM 5107; PSUC_FEM 86130, 86141, 86148, 86266 (UCFC). USA:Texas:Brazos Co.: 1 female. PSUC_FEM 56360 (CNC).

#### Antennal coiling experiment.

After rehydration of the specimens, the rami of the flagellomeres were very flexible at their bases. After the antenna were placed in distilled water, the apical flagellomeres of both specimens curled very slightly. There was no change in the angle of the flagellomere projections or movement at their bases.

## Discussion

Branched antennae are common among various groups of insects. Many Diprionidae have pectinate and bipectinate antennae, though articulated branches have not been described (Benson 1939; Benson 1945). Some Chalcidoidea, such as Eucharitidae, have ramose antennae, though none have been reported to be capable of moving the branches (pers. comm. John Heraty 2015). *Dendrocerus* of the *halidayi* species group also have ramose antennae, though none besides *Dendrocerus mexicali* have articulations (Dessart 1999).

This ramose flagellomere increases the surface area of the antenna, which could aid males in detecting female pheromones. Although nothing is known of *Dendrocerus mexicali* mating behavior, male *Dendrocerus carpenteri* have been shown to be attracted to sex pheromones released by the females (Schwörer et al. 1999). Heavy antennation during courtship has been observed, which implies the possible presence of chemosensilla on the antenna (Liebscher 1972).

Dessart postulated that the wrinkled regions at the bases of the male antennal branches were points of movement, which is extremely likely given the high resilin content of the cuticle that we found there (Figure 2) (Dessart 1999). This evolutionary novelty might allow the branches to fold, preventing the ramose antenna from getting caught on obstacles, allowing the wasp entry into a confined space, or as a mechanical defense against breakage. Hymenoptera do not have antennal pulsatory organs, but they can change the hemolymph pressure in their antenna indirectly through movements of their pharynx (Matus and Pass 1999). This movement may be controlled by the wasp via hemolymph pressure changes and hydraulics acting antagonistically against the the resilin at the base of the branch, though it may only be a passive movement of the branches when external force is applied. We replicated the Steiner et al. (2010) antennal coiling experiment to test whether the branches might be operated hydraulically and directly by the insect. Our results offer no evidence for hydraulic movement, but this could be due to damaged specimens or a more complicated mechanism.

## Author contributions

Conceived the project: IM, KNB. Character concept generation, semantic statement generation, specimen visualization and creation of plates: KNB, IM. Wrote the manuscript: KNB, IM, ARD. Commented on the final stage of the manuscript: IM, ARD.

## Acknowledgments

We would like to thank Carolyn Trietsch for creating semantic statements to describe many phenotypes and character states applicable to Ceraphronoidea. We would like to thank Lubomír Masner from the Canadian National Collection for his mentorship and access to specimens. We would like to thank the Penn State Microscopy and Cytometry Facility - University Park, PA for access to the confocal laser microscopes. This material is based upon work supported by the U. S. National Science Foundation, under Grant Numbers DBI-0850223, DBI-1356381, and DEB-1353252. Any opinions, findings, and conclusions or recommendations expressed in this material are those of the author(s) and do not necessarily reflect the views of the National Science Foundation.

## Supplementary Material

XML Treatment for Dendrocerus mexicali

## Supplementary materials

Supplementary material 1 Table 1S. Phenotype descriptions expressed as semantic statements Data type: Excel file This dataset is made available under the Open Database License (http://opendatacommons.org/licenses/odbl/1.0/). The Open Database License (ODbL) is a license agreement intended to allow users to freely share, modify, and use this Dataset while maintaining this same freedom for others, provided that the original source and author(s) are credited.Kyle N. Burks, István Mikó, Andrew R. Deans

Supplementary material 2 Table 2S. Phenotype descriptions expressed as semantic statements Data type: Excel file This dataset is made available under the Open Database License (http://opendatacommons.org/licenses/odbl/1.0/). The Open Database License (ODbL) is a license agreement intended to allow users to freely share, modify, and use this Dataset while maintaining this same freedom for others, provided that the original source and author(s) are credited.Kyle N. Burks, István Mikó, Andrew R. Deans

Supplementary material 3 Semantic statements for the taxonomic treatment of Dendrocerus mexicali Data type: OWL file This dataset is made available under the Open Database License (http://opendatacommons.org/licenses/odbl/1.0/). The Open Database License (ODbL) is a license agreement intended to allow users to freely share, modify, and use this Dataset while maintaining this same freedom for others, provided that the original source and author(s) are credited.Kyle N. Burks, István Mikó, Andrew R. Deans
